# Predicting suitable areas for *Metcalfa pruinosa* (Hemiptera: Flatidae) under climate change and implications for management

**DOI:** 10.1093/jisesa/ieae053

**Published:** 2024-05-08

**Authors:** Zhengxue Zhao, Lin Yang, Jiankun Long, Zhimin Chang, Xiangsheng Chen

**Affiliations:** Institute of Entomology, College of Agriculture, Guizhou University, Guiyang 550025, PR China; Provincial Special Key Laboratory for Development and Utilization of Insect Resources of Guizhou, College of Agriculture, Guizhou University, Guiyang 550025, PR China; Guizhou Key Laboratory for Agricultural Pest Management of Mountainous Region, College of Agriculture, Guizhou University, Guiyang 550025, PR China; Key Laboratory of High-efficiency Agricultural Plant Protection Informatization in Central Guizhou, College of Agriculture, Anshun University, Anshun 561000, PR China; Institute of Entomology, College of Agriculture, Guizhou University, Guiyang 550025, PR China; Provincial Special Key Laboratory for Development and Utilization of Insect Resources of Guizhou, College of Agriculture, Guizhou University, Guiyang 550025, PR China; Guizhou Key Laboratory for Agricultural Pest Management of Mountainous Region, College of Agriculture, Guizhou University, Guiyang 550025, PR China; Institute of Entomology, College of Agriculture, Guizhou University, Guiyang 550025, PR China; Provincial Special Key Laboratory for Development and Utilization of Insect Resources of Guizhou, College of Agriculture, Guizhou University, Guiyang 550025, PR China; Guizhou Key Laboratory for Agricultural Pest Management of Mountainous Region, College of Agriculture, Guizhou University, Guiyang 550025, PR China; Institute of Entomology, College of Agriculture, Guizhou University, Guiyang 550025, PR China; Provincial Special Key Laboratory for Development and Utilization of Insect Resources of Guizhou, College of Agriculture, Guizhou University, Guiyang 550025, PR China; Guizhou Key Laboratory for Agricultural Pest Management of Mountainous Region, College of Agriculture, Guizhou University, Guiyang 550025, PR China; Institute of Entomology, College of Agriculture, Guizhou University, Guiyang 550025, PR China; Provincial Special Key Laboratory for Development and Utilization of Insect Resources of Guizhou, College of Agriculture, Guizhou University, Guiyang 550025, PR China; Guizhou Key Laboratory for Agricultural Pest Management of Mountainous Region, College of Agriculture, Guizhou University, Guiyang 550025, PR China

**Keywords:** *Metcalfa pruinosa*, climate change, suitable area, Maxent, pest management

## Abstract

Climate change is a prominent factor reshaping the distribution of invasive species. *Metcalfa pruinosa* (Say 1830) (Hemiptera: Flatidae), native to North America, has invaded other continents and poses a serious threat to various agricultural crops and the human residential environment. Understanding the distribution of *M. pruinosa* based on climatic conditions is a critical first step to prevent its further invasion. Therefore, based on its occurrence records and associated environmental variables, a Maxent model was developed to predict suitable areas for this species in the present and future on a global scale. The model exhibited outstanding performance, with a mean area under the receiver operating characteristic curve and true skill statistic values of 0.9329 and 0.926, respectively. The model also indicated that annual precipitation (Bio12) and max temperature of the warmest month (Bio5) were the key environmental variables limiting the distribution of *M. pruinosa*. Moreover, the model revealed that the current suitable area is 1.01 × 10^7^ km^2^ worldwide, with southern China, southern Europe, and the eastern United States predicted to be the primary and highly suitable areas in the latter 2 regions. This area is expected to increase under future climate scenarios, mainly in the northern direction. The study’s findings contribute to our understanding of climate change’s impact on *M. pruinosa* distribution, and they will aid governments in developing appropriate pest management strategies, including global monitoring and strict quarantine measures.

## Introduction

Invasive species refers to introduced species that advance without direct human assistance; threaten natural or seminatural habitats outside their natural range; and have social, environmental, or economic impacts (McNeely 2001), such as threatening human health (Mazza et al. 2014), species extinction (Bellard et al. 2016), and rising economic costs (Diagne et al. 2021). Unfortunately, climate change may lead to the expansion of geographical ranges of invasive species (Huang et al. 2019, Liu and Shi 2020, Ramasamy et al. 2021), which is expected to exacerbate the damage. Invasive species are often extremely difficult to eradicate once established (Rejmánek et al. 2005). Hence, preventing their introduction is the most cost-effective form of management (Gallien et al. 2012). Given their potentially substantial negative impact, suitable tools should be developed to help prevent the introduction of invasive species.

*Metcalfa pruinosa* (Hemiptera: Flatidae), the citrus flatid planthopper, is native to North America (Metcalf and Bruner 1948); however, it has invaded several regions outside of its native range (Zangheri and Donadini 1980, Kim et al. 2011, Mitrea 2018, Świerczewski et al. 2023). *Metcalfa pruinosa* is a prominent pest that causes multifaceted harm. For example, in South Korea, it has caused severe economic damage to various agricultural crops, including pepper, apple, and bean, by direct feeding and substantial production of waxy secretions and honeydew (Kim et al. 2011). Therefore, *M. pruinosa* is listed as a quarantine pest by the National Plant Quarantine Service in South Korea. Furthermore, the increase in *M. pruinosa* populations has adversely affected the human residential environment (Byeon et al. 2017a). *Metcalfa pruinosa* also acts as a vector for harmful bacteria (e.g., *Pseudomonas syringae* pv. *actinidiae*) or phytoplasmas (e.g., Aster yellows phytoplasmas) (Donati et al. 2017, Mergenthaler et al. 2020).

As highlighted in a previous study (Byeon et al. 2017a), suitable habitats of *M. pruinosa*, as determined by climatic conditions, are a critical first step in developing effective control strategies. Several previous studies have focused on potentially suitable areas for *M. pruinosa* nationally (South Korea and Austria) (Strauss 2010, Byeon et al. 2017b, 2018, Kim et al. 2019, Lee et al. 2019). Only one study has predicted global suitable areas in the present and future to be distributed across almost all continents, with specific remarkable regions such as eastern North America and the southern half of Africa, and that these areas would expand toward northern regions in the future (Byeon et al. 2017a). Notably, the projection results were obtained using the CLIMEX model without performance evaluation. The CLIMEX model is a mechanistic species distribution model, and the output results are susceptible to changes in several parameters, i.e., limiting low temperature, high temperature, and low soil moisture, requiring extensive research and data collection (Taylor and Kumar 2012). Unfortunately, this was not rigorously performed in the previous study owing to insufficient phenology data, which also used the future climate from the A1B scenario of the Special Report on Emissions Scenarios to predict future distribution (Byeon et al. 2017a). This scenario is outdated, as it was published in 2000. The latest future climate scenarios provided by the Coupled Model Intercomparison Project Phase 6 (CMIP6) were released recently and extensively used in modeling the future distribution of invasive pests (Zhang et al. 2021, 2022, Garcia et al. 2022). Consequently, based on the above description, it must be acknowledged that the present and future distribution of *M. pruinosa* is poorly understood and urgently needs investigation. Complete phenology data for *M. pruinosa* (i.e., temperature and moisture preferences and wet, cold, heat, and dry stresses) are still lacking, making mechanistic models, such as CLIMEX, unsuitable; thus, alternative models should be considered.

Correlative species distribution models use associations between known occurrence records of species and environmental conditions to predict potential geographic distributions of species (Feng et al. 2019, Feng 2023). Several models, such as maximum entropy (Maxent), random forests, and boosted regression trees, have been developed. These models allow the selection of an appropriate model according to the purpose of the study. Among them, the Maxent model had numerous advantages over the others, such as the efficient handling of complex interactions between response and predictor variables (Elith et al. 2006, 2011), outstanding performance (Phillips et al. 2006), and extreme simplicity of use (Fourcade et al. 2014). Consequently, many studies have extensively applied the Maxent model to predict the geographical distribution range of invasive species, particularly insects (Zhu et al. 2016, Wang et al. 2017, Tang et al. 2019, Gao et al. 2022). The suitable areas projected by the Maxent model are useful for developing management measures to control invasive species.

This study aimed to develop a Maxent model to project global suitable areas for *M. pruinosa* in the present and future. We solve the following 3 problems: (i) What are the main factors affecting species distribution? (ii) What is the distribution pattern of suitable areas in the present? (iii) How would the distribution of suitable areas change in the future?

## Materials and Methods

### Species Distribution Data

**Fig. 1.**
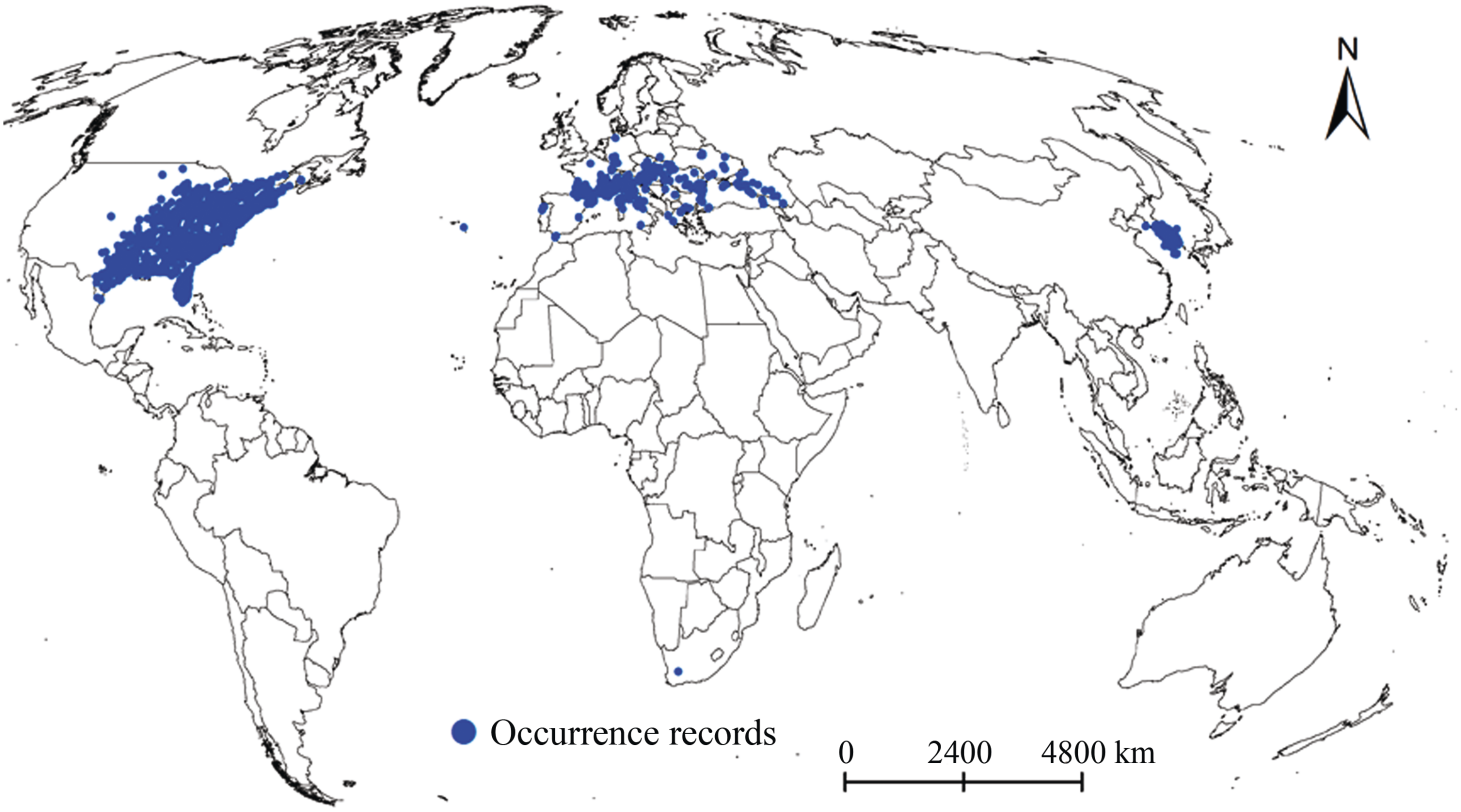
Occurrence records of *Metcalfa pruinosa* on a global scale.

Initial occurrence records of *M. pruinosa* were obtained from the Global Biodiversity Information Facility (https://www.gbif.org/) and the literature. These data were filtered by removing occurrence records with errors in biological collection data (e.g., sea coordinates) and high coordinate uncertainty (>20 km) using the CoordinateCleaner Package in R 4.2.1 (Zizka et al. 2019). Literature records lacking latitude and longitude data were georeferenced using Google Earth. Studies have indicated that sampling bias in occurrence records can affect the prediction results of species distribution models (Kramer-Schadt et al. 2013, Boria et al. 2014). To solve this problem, we conducted spatial thinning for a distance of 20 km using the spThin package in R 4.2.1 (Aiello-Lammens et al. 2015). Finally, 1,471 occurrence records were included in subsequent analysis (Fig. 1; Supplementary Table S1).

### Environmental Variables

The current and future environmental variables were obtained from the WorldClim website (https://www.worldclim.org) at a spatial resolution of 5 arc min. The current variables represented in the 1970–2000 period consisted of 19 bioclimatic variables and one altitude data. Pearson’s correlation analysis for the environmental variable values of the occurrence records was first performed using SPSS 25 to reduce the influence of the collinearity of variables on the prediction results (Supplementary Table S2). Furthermore, it retained one between 2 highly correlated variables (*r* ≥ 0.85). Next, the variance inflation factor (VIF) of the retained variables was calculated (Supplementary Table S2), and those with the highest VIF value until less than 5 were omitted. Finally, 7 environmental variables: mean diurnal range (Bio2) (1–21.73 °C), isothermally (Bio3) (9.06–100 °C), max temperature of warmest month (Bio5) (−29.7 to 48.26°C), mean temperature of driest quarter (Bio9) (−54.89 to 37.6°C), annual precipitation (Bio12) (0–11,191 mm), precipitation seasonality (Bio15) (0–229.377 mm), and altitude were selected (−415 to 6,574 m).

Future bioclimatic variables provided by CMIP6 during the periods of 2041–2060, 2061–2080, and 2081–2100 were used. Furthermore, 2 shared socioeconomic pathways representing low and high-emission scenarios, SSP126 and SSP585, were selected. The bioclimatic variables were obtained by calculating the mean of the CanESM5, IPSL-CM6A-LR, and MIROC6 model data to reduce future climate uncertainty.

### Maxent Model

The Maxent model was optimized by choosing the feature-type combination and regularization multiplier values from 0.5 to 4, with increments of 0.5, using L, H, LQ, LQH, LQHP, and LQHPT (L, linear; Q, quadratic; H, hinge; P, product; and T, threshold). Furthermore, the ENMeval package in R 4.2.1 was applied to select the 2 optimal parameters based on the lowest score of the corrected Akaike information criterion using the checkerboard2 approach (Muscarella et al. 2014). The checkerboard2 approach generates checkerboard grids across the study extent, partitioning data into *k* = 4 spatial groups by hierarchically aggregating the input raster at 2 scales. Finally, a regularized multiplier of 0.5 and a feature combination of LQHPT were obtained. Furthermore, the optimal model in Maxent (v3.4.4) software was performed with 5-fold cross-validation, 10,000 background points, and cloglog output. To validate the performance of these models, the area under the receiver operating characteristic curve (AUC) and true skill statistic (TSS) were used; the models were considered excellent when their values were >0.9 and 0.8, respectively (Ben Rais Lasram et al. 2010, Bogawski et al. 2019).

### Suitable areas and Their Change

An area was identified as suitable or unsuitable based on the threshold of maximum training sensitivity plus specificity (0.25). Furthermore, the suitable areas were classified into lowly (0.25–0.4), moderately (0.4–0.6), and highly (0.6–1) suitable areas (Wei et al. 2020, Wang et al. 2021). The distributional changes in these areas under climate change were obtained using the SDMtoolbox in ArcGIS 10.7, which identified the spatial patterns and the suitable area under contraction, expansion, and unchanged (Brown 2014).

## Results

### Model Performance

The values of the 2 model evaluation metrics, AUC and TSS obtained, were >0.9 for each replicate (Table 1), using regularized multiplier = 0.5 and feature combination = LQHPT. The mean AUC and TSS values of 5 replicates were 0.9329 and 0.926, respectively (Table 1), which indicated that the model built in this study was excellent.

**Table 1.** AUC and TSS values of the Maxent model

Replicates	AUC	TSS
1	0.9316	0.9271
2	0.9337	0.9173
3	0.9329	0.9363
4	0.9355	0.9295
5	0.9311	0.9199
Mean	0.9329	0.9260

### Importance of Environmental Variables on Species Distribution

**Fig. 2.**
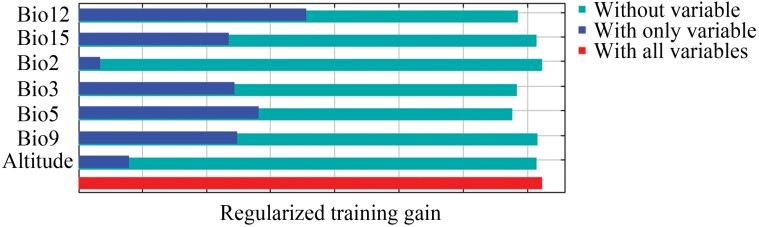
Relative importance of the 7 environmental variables determined using the jackknife test.

The relative importance of 7 environmental variables that influence the distribution of *M. pruinosa* was determined based on the jackknife test (Fig. 2). The significance of each variable was different, with Bio12 and Bio5 having the greatest effect (Fig. 2). The importance of Bio3, Bio9, and Bio15 was almost similar, while that of the Bio2 and altitude was relatively low (Fig. 2).

**Fig. 3.**
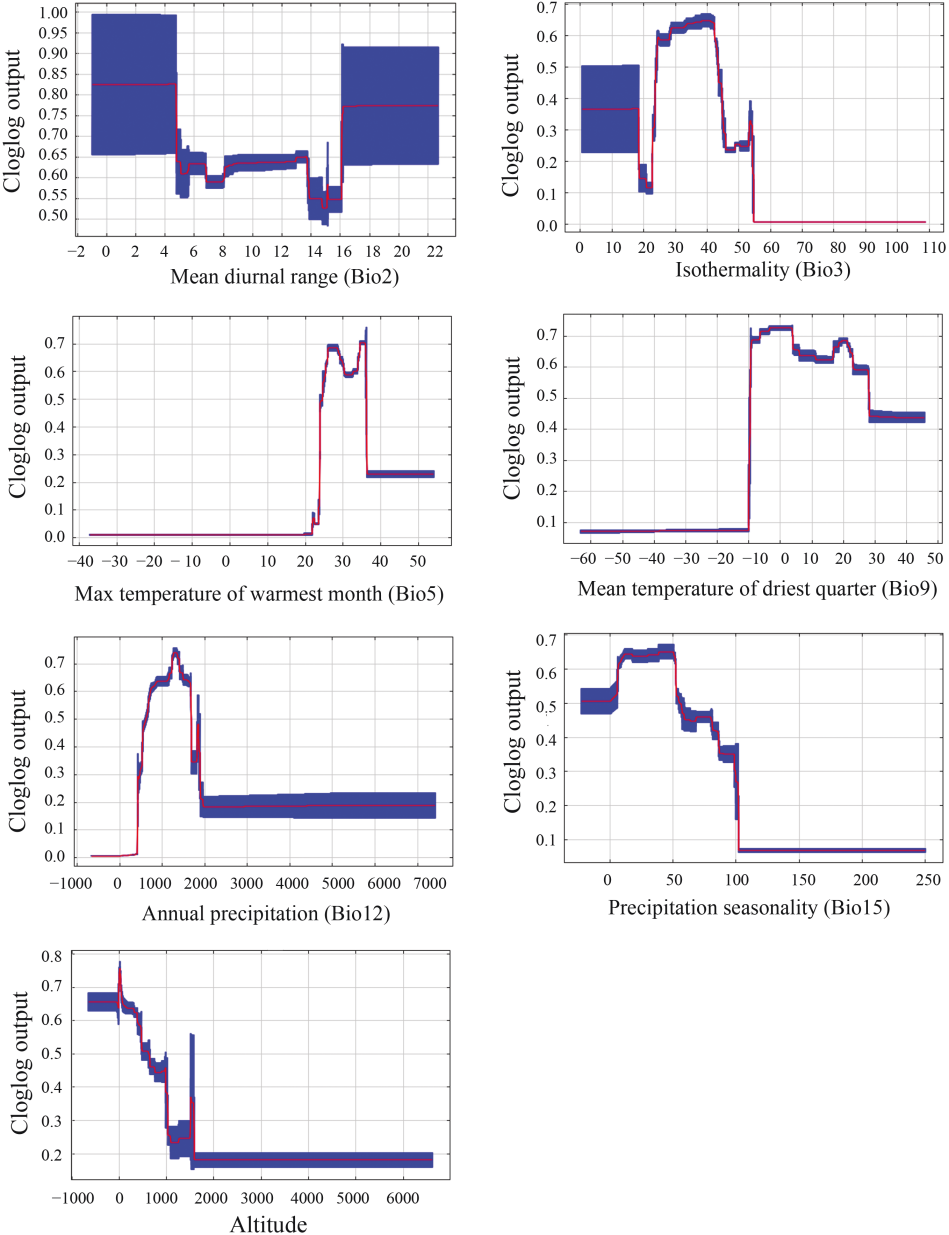
Response curves indicating changes in the presence probability along with the environmental variables.

The response curve revealed probability changes of *M. pruinosa* presence as each environmental variable changed. Ranges of each variable contributed to high probability of presence (Fig. 3). Specifically, the high probability occurred at −0.97 to 22.68°C of Bio2, 28.28–42.28 °C of Bio3, 24.65–36.15 °C of Bio5, −9.46 to 22.82°C of Bio9, 725.54–1,672.26 mm of Bio12, 6.45–51.99 mm of Bio15, and −415 to 380.41 m of altitude.

### Current Suitable Areas

**Fig. 4.**
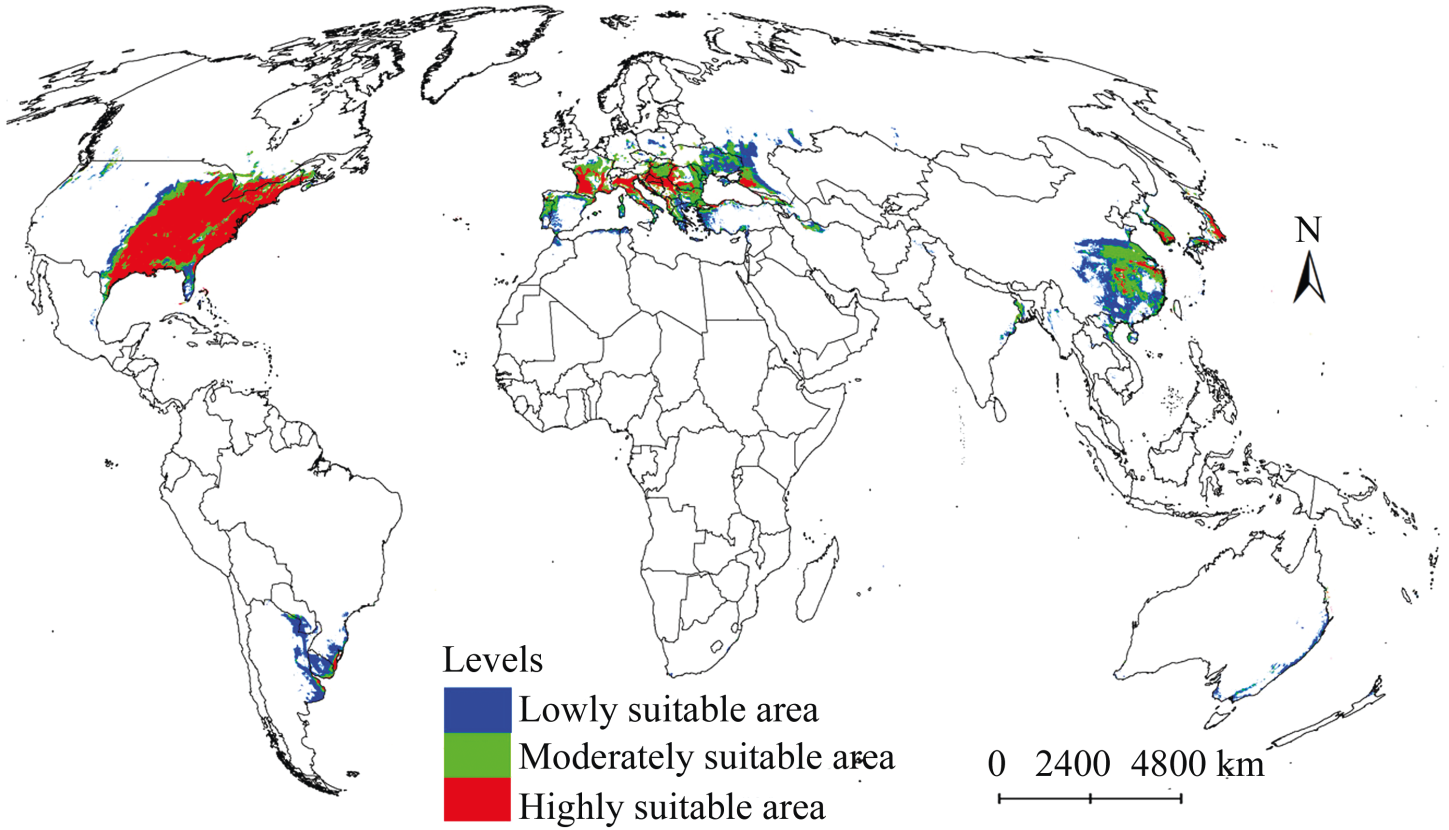
Currently suitable areas for *Metcalfa pruinosa* on a global scale.

The Maxent model predicted that the total suitable areas for *M. pruinosa* include a 1.01 × 10^7^ km^2^ area worldwide (Table 2), mainly distributed in southern China, southern Europe, and the eastern United States (Fig. 4). In addition, some areas were detected in South Korea and Japan and several other countries in South America, such as Brazil, Argentina, Uruguay, and Paraguay (Fig. 4). Some were identified in other regions, such as the southeast coast of Australia and Southeast Canada. Among the 3 levels of suitable areas, the highly suitable area was the largest, reaching 3.94 × 10^6^ km^2^, followed by the lowly suitable area at 3.33 × 10^6^ km^2^ and the moderately suitable area at 2.79 × 10^6^ km^2^ (Table 2). Highly suitable areas were mainly distributed in the eastern United States and southern European countries (France, Italy, Croatia, Romania, and Serbia), whereas moderately suitable areas were mainly concentrated in China, Hungary, Bulgaria, and Romania (Fig. 4). The distribution of lowly suitable areas was more dispersed compared with the other 2 types, with notable occurrences in Ukraine, southwestern Russia, southern China, the United States, South America, and Australia (Fig. 4).

**Table 2.** Areas with the suitability for *Metcalfa pruinosa* in the current and future (km^2^)

	Current	2041–2060		2061–2080		2081–2100	
		SSP126	SSP585	SSP126	SSP585	SSP126	SSP585
Total	1.01 × 10^7^	1.23 × 10^7^	1.29 × 10^7^	1.233 × 10^7^	1.25 × 10^7^	1.232 × 10^7^	1.12 × 10^7^
Low	3.33 × 10^6^	3.52 × 10^6^	3.75 × 10^6^	3.46 × 10^6^	4.46 × 10^6^	3.44 × 10^6^	4.83 × 10^6^
Moderate	2.79 × 10^6^	4.09 × 10^6^	4.40 × 10^6^	4.09 × 10^6^	4.77 × 10^6^	4.05 × 10^6^	4.31 × 10^6^
High	3.94 × 10^6^	4.69 × 10^6^	4.72 × 10^6^	4.78 × 10^6^	3.24 × 10^6^	4.83 × 10^6^	2.03 × 10^6^

### Future Suitable Areas and Changes

**Fig. 5.**
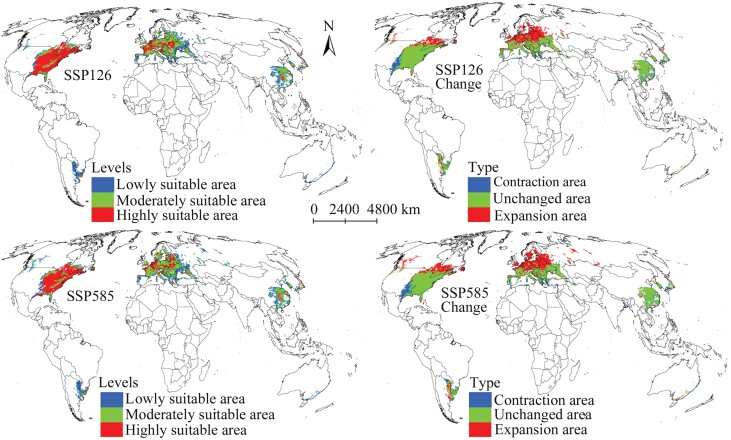
Suitable areas for *Metcalfa pruinosa* and their changes in 2041–2060 on a global scale.

Under the scenario SSP126 in 2041–2060, the total suitable area of *M. pruinosa* expanded to 1.23 × 10^7^ km^2^; this trend was also observed in the highly, moderately, and lowly suitable areas (Table 2). Highly suitable areas continued to be concentrated in southern Europe and the eastern United States (Fig. 5). Compared with the current period, the expansive suitability area reached 4.0 × 10^6^ km^2^ (Table 3), mainly in southeastern Canada and northern Europe, indicating that the suitable area would move further north (Fig. 5). Furthermore, a small range of expansive suitability areas were noted in some regions such as southern China and eastern Argentina. The contractive suitability area was 1.4 × 10^6^ km^2^, concentrated in the southwest of the United States, whereas the unchanged suitability area was the largest, reaching 8.36 × 10^6^ km^2^ (Table 3). Under the climate scenario SSP585, the total suitable area for *M. pruinosa* is expected to increase relative to the current period (Table 2). Consistent with the results of the climate scenario SSP126, the expansion of suitable areas occurred mainly in southeastern Canada and northern Europe (Fig. 5).

**Table 3.** Change in future suitable areas for *Metcalfa pruinosa* (km^2^)

	2041–2060		2061–2080		2081–2100	
	SSP126	SSP585	SSP126	SSP585	SSP126	SSP585
Expansion	4.0 × 10^6^	4.92 × 10^6^	4.12 × 10^6^	6.18 × 10^6^	4.05 × 10^6^	7.11 × 10^6^
Contraction	1.4 × 10^6^	1.75 × 10^6^	1.48 × 10^6^	3.14 × 10^6^	1.44 × 10^6^	5.65 × 10^6^
Unchanged	8.3 × 10^6^	7.96 × 10^6^	8.23 × 10^6^	6.29 × 10^6^	8.26 × 10^6^	4.06 × 10^6^

**Fig. 6.**
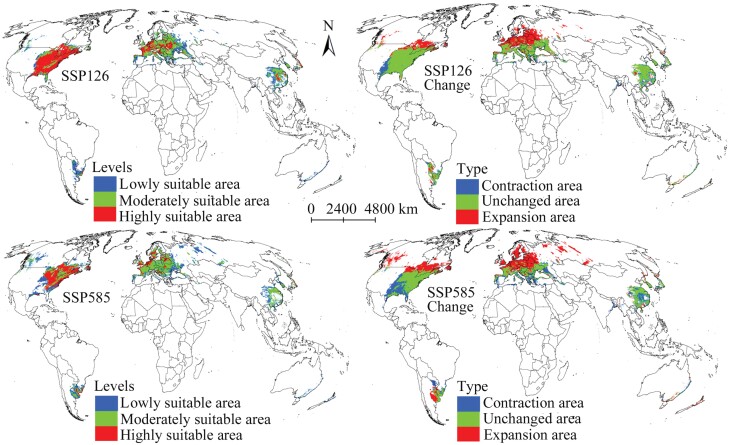
Suitable areas for *Metcalfa pruinosa* and their changes in 2061–2080 on a global scale.

The total suitable areas and the 3 levels of suitable areas for *M. pruinosa* under the future climate scenario SSP126 in 2061–2080 were conspicuously more prominent than those in the present (Table 2). The distribution pattern and spatial change in suitable areas under this future climate scenario were similar to that under the climate scenario SSP126 in 2041–2060 (Figs. 5 and 6). Under the climate scenario SSP585, the total suitable area continued to increase and was predicted to cover 1.25 × 10^7^ km^2^ (Table 2). Moreover, the projection results indicated that the moderately and lowly suitable areas also increased, but the highly suitable area decreased. The projection results revealed that the suitable area would continue to expand toward the north compared with today (Fig. 6). Additionally, expansive and contractive suitability areas are projected to occupy a more extensive distribution range compared with 2041–2060, whereas the unchanged suitability areas were projected to reduce (Fig. 6; Table 3).

**Fig. 7.**
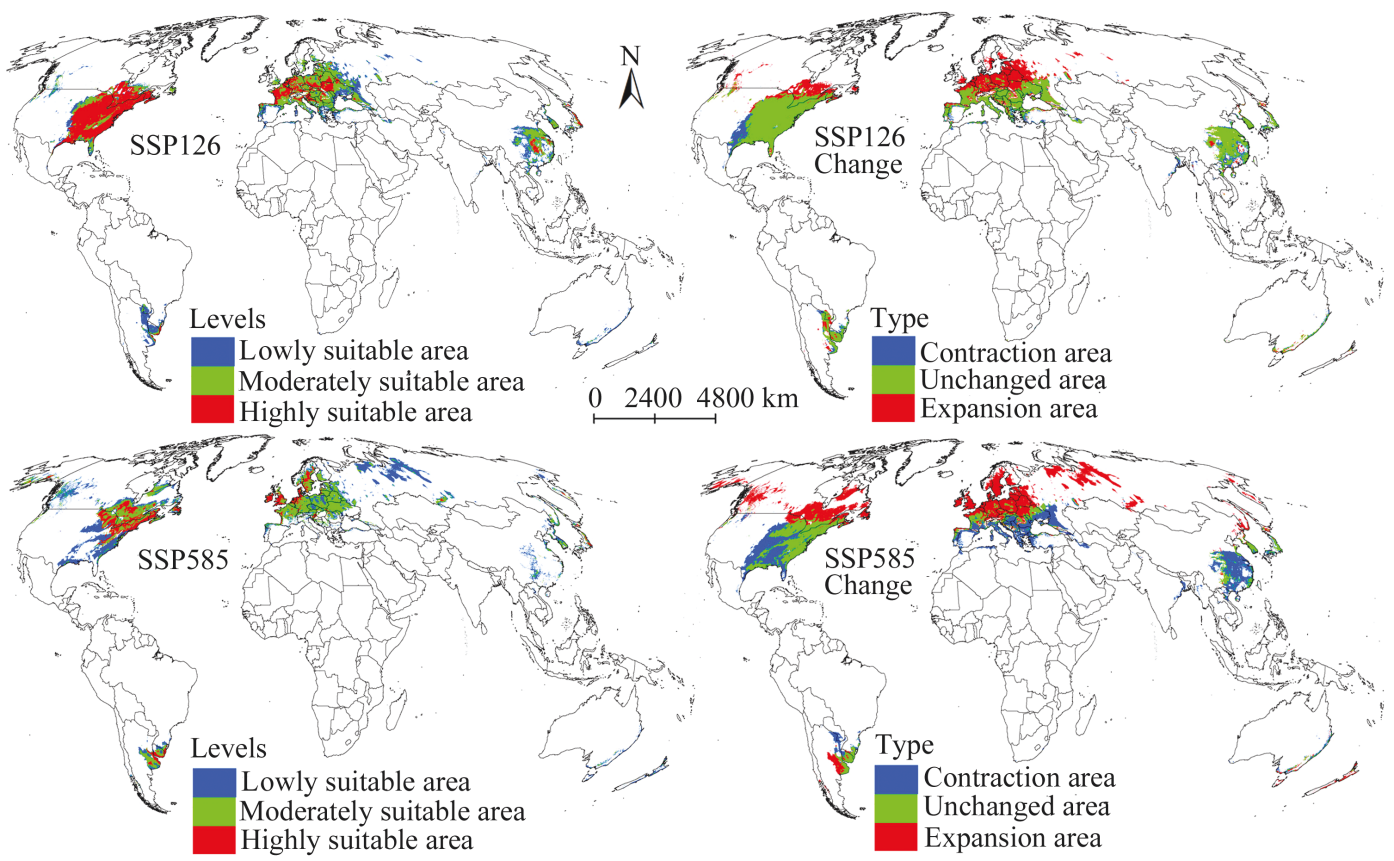
Suitable areas for *Metcalfa pruinosa* and their changes in 2081–2100 on a global scale.

Predicted total suitability areas for *M. pruinosa* under the climate scenario SSP126 in 2081–2100 were also more prominent than those at present but lesser than those of the scenarios in 2061–2080 (Table 2). The results also suggested that the suitable areas of the 3 levels will increase compared with those under current environmental conditions. The expansive suitability areas reached 4.05 × 10^6^ km^2^ (Table 3). They were primarily distributed in southeastern Canada and northern Europe (Fig. 7). The unchanged suitability area was projected to cover 8.25 × 10^6^ km^2^, mainly in southern China, southern Europe, and the eastern United States (Fig. 7). Under the climate scenario SSP585, the highly suitable area would be smaller than that at present; however, the moderately and lowly suitable areas is expected to increase (Table 2). Furthermore, the expansive suitability area was the largest under all future climate scenarios at 7.11 × 10^6^ km^2^ (Table 3). The projection results also demonstrated many suitable areas for *M. pruinosa* in western Canada and western Russia (Fig. 7), indicating that this invasive species has a much broader expansion to the north.

## Discussion

This study predicted the current and future suitable areas for *M. pruinosa* worldwide using the Maxent model, with the main environmental variables that affected this distribution being Bio12 and Bio5. Therefore, these results highlighted the key roles of temperature and precipitation variables together, aligning with a previous study on modeling suitable areas for this pest in South Korea (Kim et al. 2019). Moreover, similar results have been found in other invasive pests, including *Daktulosphaira vitifoliae* (Wei et al. 2021), *Spodoptera frugiperda* (Ramasamy et al. 2021), and *Drosophila suzukii* (Dos Santos et al. 2017). Furthermore, based on the response curve, the range values of the 2 main environmental variables were also obtained when *M. pruinosa* had high suitability, with 725.54–1,672.26 mm of Bio12 and 24.65–36.15 °C of Bio5. These values reflect the optimal climatic conditions for *M. pruinosa* population growth; however, the range of these climatic variables may not accurately represent environmental requirements, given the model’s correlative nature, and could include unobserved interactions with biotic factors or abiotic confounders.

The distribution range of the predicted suitable areas for *M. pruinosa* was markedly more considerable than the observed distributions. At present, some regions, such as southern China, eastern Argentina, and central and southern Japan, have not recorded *M. pruinosa* but are predicted to be suitable habitats for this pest (Figs. 1 and 4) and, therefore, should be concerned. The prediction results by the model also showed that although the highly suitable areas are concentrated in the eastern United States and southern Europe, the former occupy a wider distribution range (Fig. 4), which reflects that the climatic conditions in the eastern United States are more suitable for *M. pruinosa* survival. A few regions of China, South Korea, and Japan were also predicted to be highly suitable areas. *Metcalfa pruinosa* has been documented, and outbreaks have occurred in South Korea (Kim et al. 2011, 2019, 2020), but China and Japan have not reported this pest so far. Interestingly, the prediction results showed that the total suitable and highly suitable areas in China and Japan are only distributed in the southern, central, and eastern regions of the respective mainland, which are adjacent to South Korea (Fig. 4). Therefore, suitable areas located in China and Japan were most probably invaded by *M. pruinosa* from South Korea. Proactive management strategies such as monitoring, surveillance, and strict quarantine measures should be implemented through the local governments of the 2 countries.

Climate change affects suitable areas for invasive pests (Abou-Shaara et al. 2021, Tepa-Yotto et al. 2021, Xue et al. 2022); such changes should be considered when developing pest management strategies. This study predicted that the suitable areas for *M. pruinosa* will expand under all climate scenarios, indicating a significant increase in its invasive ability. Previous studies that modeled the habitat suitability of other invasive pests reported similar results (Neven et al. 2018, Liu and Shi 2020, Chen et al. 2023). This study also indicated that the suitable areas for *M. pruinosa* will mainly expand to the northern regions (higher latitude), particularly significantly under the climate scenario SSP585 in 2081–2100. This result can be attributed to the change in climate conditions from unsuitable to suitable in the northern regions for the population growth of *M. pruinosa* in the future. Nevertheless, suitable areas are not expected to expand in all regions in the future, and a typical example is the eastern United States. In future climate scenarios, the suitable area in the eastern United States is expected to decrease, especially under the climate scenario SSP585 in 2081–2100 (Fig. 7). The reduction of the suitable regions will result in many benefits, such as reducing investment in pest management and increasing opportunities to reverse the current invasion landscape locally (Wei et al. 2018, 2019). Obtaining the spatial distribution pattern of expansion, contraction, and unchanged suitability areas under future climate change can provide more effective guidance for implementing pest management measures. Specifically, appropriate pest management measures should be continuously applied in areas of unchanged suitability to achieve lasting control. It is the need of the hour to improve monitoring and implement strict quarantine measures in the expansion suitability area to prohibit *M. pruinosa* from invading other regions. Furthermore, we need not focus on the contraction suitability area in the future as it is expected to become unsuitable for the survival of *M. pruinosa*.

Our predictive results differ from a previous global scale study (Byeon et al. 2017a), which predicted broader current suitable areas compared with the present study, with some suitable regions, such as southern Africa and northeastern China. Additionally, we noted that the future suitable areas in China were larger in the previous study than in the present study. The discrepancies in results may be attributed to spatial distribution patterns, variations in environmental variables, and different models. Nevertheless, the present study’s predictions hold higher credibility due to certain shortcomings in the previous study.

Although this study establishes a model exhibiting outstanding performance, there are several limitations to it. The occurrence records of species and environmental variables are the basic data needed for constructing species distribution models and are related to the performance of models (Hernandez et al. 2006, Wisz et al. 2008, Feeley and Silman 2011). Usually, inaccurate data are removed from the initial species occurrence records compiled from various sources (e.g., museums, literature, and related databases), and only accurate data are used for constructing species distribution models, as was done in this study. However, this reduces the sample size and negatively influences model performance (McPherson et al. 2004, Hernandez et al. 2006). *Metcalfa pruinosa* is a herbivorous species that feeds on a variety of plants (Bagnoli and Lucchi 2000, Wilson and Lucchi 2000, Seo et al. 2019). Therefore, the distribution of host plants inevitably affects its range. Unfortunately, data on the spatial distribution of host plants are currently lacking, which results in this information not being considered in the model. In addition, false absence points in the background points of species distribution models can affect prediction results (Lobo et al. 2010). Therefore, to further improve the model’s accuracy, these limitations should be considered in the Maxent model in future research.

## Conclusions

This study developed an excellent Maxent model to predict the present and future suitable areas for *M. pruinosa* across the globe. The results emphasized that temperature and precipitation were the main environmental variables in determining the distribution of *M. pruinosa*. The currently suitable areas for *M. pruinosa* are mainly located in southern China, southern Europe, and the eastern United States, and the latter 2 regions were also identified as being highly suitable for this invasive pest. In future climate scenarios, the suitable area of *M. pruinosa* is expected to expand mainly northward compared with the current time, especially under the SSP585 scenario in 2081–2100. The predictive results of this study provide insights into the risk posed by introductions of *M. pruinosa* and the theoretical guidance framework for managing it.

## Supplementary Material

ieae053_suppl_Supplementary_Tables_S1

ieae053_suppl_Supplementary_Tables_S2
